# In Vitro Activities of Oxazolidinone Antibiotics Alone and in Combination with C-TEMPO against Methicillin-Resistant *Staphylococcus aureus* Biofilms

**DOI:** 10.3390/antibiotics12121706

**Published:** 2023-12-07

**Authors:** Audrey R. N. Ndukwe, Jilong Qin, Sandra Wiedbrauk, Nathan R. B. Boase, Kathryn E. Fairfull-Smith, Makrina Totsika

**Affiliations:** 1 Centre for Immunology and Infection Control, School of Biomedical Sciences, Faculty of Health, Queensland University of Technology, Brisbane, QLD 4001, Australia; audrey.ndukwe@hdr.qut.edu.au (A.R.N.N.); jilong.qin@qut.edu.au (J.Q.); 2 School of Chemistry and Physics, Queensland University of Technology, Brisbane, QLD 4001, Australia; sandra.wiedbrauk@qut.edu.au (S.W.); nathan.boase@qut.edu.au (N.R.B.B.); k.fairfull-smith@qut.edu.au (K.E.F.-S.); 3 Centre for Materials Science, Queensland University of Technology, Brisbane, QLD 4001, Australia; 4 Max Planck Queensland Centre, Queensland University of Technology, Brisbane, QLD 4001, Australia

**Keywords:** MRSA, biofilm, antimicrobial, oxazolidinone, nitroxide, *Staphylococcus aureus*, antibiofilm, biofilm eradication, MBEC

## Abstract

Infections caused by methicillin-resistant *Staphylococcus aureus* (MRSA) are a global health concern. The propensity of MRSA to form biofilms is a significant contributor to its pathogenicity. Strategies to treat biofilms often involve small molecules that disperse the biofilm into planktonic cells. Linezolid and, by extension, theoxazolidinones have been developed to treat infections caused by Gram-positive bacteria such as MRSA. However, the clinical development of these antibiotics has mainly assessed the susceptibility of planktonic cells to the drug. Previous studies evaluating the anti-biofilm activity of theoxazolidinones have mainly focused on the biofilm inhibition of *Enterococcus faecalis* and methicillin-sensitive *Staphylococcus aureus*, with only a few studies investigating the activity of oxazolidinones for eradicating established biofilms for these species. Very little is known about the ability of oxazolidinones to eradicate MRSA biofilms. In this work, five oxazolidinones were assessed against MRSA biofilms using a minimum biofilm eradication concentration (MBEC) assay. All oxazolidinones had inherent antibiofilm activity. However, only ranbezolid could completely eradicate MRSA biofilms at clinically relevant concentrations. The susceptibility of the MRSA biofilms to ranbezolid was synergistically enhanced by coadministration with the nitroxide biofilm dispersal agent C-TEMPO. We presume that ranbezolid acts as a dual warhead drug, which combines the mechanism of action of the oxazolidinones with a nitric oxide donor or cytotoxic drug.

## 1. Introduction

Bacterial infections caused by methicillin-resistant *Staphylococcus aureus* (MRSA) are a major public health issue, accounting for 13–74% of *S. aureus* infections worldwide [1,2,3,4]. In the US alone, approximately 7 million people are hospitalised due to MRSA infections each year, representing a USD 2.7 million financial burden [5,6]. MRSA is a prominent pathogen in skin and soft tissue infections, bacteremia, pneumonia, septic arthritis, osteomyelitis, endocarditis and necrotizing fasciitis and is difficult to treat [7,8]. Exacerbating the problem further is the ability of MRSA to form biofilms, which is a significant contributor to chronic infections such as skin lesions and urinary tract infections [9,10,11]. A biofilm consists of a community of bacteria typically adhered to a biotic or abiotic surface and that is encased in an extracellular polymeric substance containing proteins, carbohydrates and extracellular DNA [12]. Small molecules such as nitroxides have been shown to disperse biofilms into planktonic cells [13]. However, as these dispersed cells are not killed, they can move to a distal site and re-form the biofilm, causing relapsing infections [14]. Considering the significant toll of MRSA on public health globally, the World Health Organisation (WHO) has identified MRSA as a “high priority” pathogen for which the development of new treatments is urgently required [15].

The first-in-class oxazolidinone antibiotic, linezolid, is an important antibiotic in the treatment of Gram-positive pathogens and has demonstrated potent activity against MRSA infections [16,17]. From the 1960s to the 1980s, antibiotic development focused heavily on Gram-negative pathogens. The oxazolidinones were first discovered in the late 1980s as a response to the rising rates of resistance. At that time, vancomycin had become the only antibiotic that could be used to treat MRSA, and vancomycin resistance was beginning to emerge [18]. Linezolid was approved by the US Food and Drug Administration (FDA) for clinical use in 2000 to treat nosocomial pneumonia and skin and soft tissue infections caused by MRSA [19]. The only additional oxazolidinone approved for clinical use to date is tedizolid, which is also used to treat skin infections [20]. Other oxazolidinones such as ranbezolid, radezolid, contezolid (MRX-4), sutezolid and delpazolid (LCB01-0371) have all reached phase 2 clinical trials, while cadazolid reached phase 3 trials [21,22,23,24,25,26,27,28,29]. Depazolid and sutezolid are still undergoing phase 2 trials, but there is currently no indication that phase 3 or 4 trials are underway for the other oxazolidinones listed [27,28,30]. Interestingly, sutezolid was originally in development alongside linezolid for Gram-positive infections such as MRSA but was found to have superior anti-tuberculosis activity [31]. While depazolid was developed decades later, it has followed a similar development path to sutezolid as it was originally developed to treat MRSA and other Gram-positive infections such as penicillin-resistant *Streptococcus pneumoniae* but was also found to be a superior anti-tuberculosis agent [32,33]. A number of other oxazolidinones have also been clinically tested over the years, but development was halted due to either poor safety profiles, low solubility, poor pharmacokinetic profiles, or not having significant differentiation in activity in comparison to linezolid [34]. Interestingly, based on the available clinical trial data, not all the clinical trials included chronic patient cohorts [21,23,24,25,26,27,28,30,35].

The clinical development of most antibiotics, including oxazolidinones, involves determining antibacterial activity (bactericidal or bacteriostatic) against bacteria growing in the planktonic state. Testing activity against biofilms is not routinely performed during drug development despite the fact that biofilms are significant contributors to pathogenicity and virulence [36]. In comparison to planktonic bacteria, biofilms have been shown to be up to 1000 times more tolerant to antibiotics [9,37,38]. It has been reported that MRSA is prevalent in healthcare settings, and these infections are often associated with biofilms, resulting in treatment failure and mortality [39,40,41]. From a resistance standpoint, linezolid and oxazolidinones, by extension, are ideal candidates for future development for biofilm infections because there are limited reports of resistance to linezolid, with >98% of *S. aureus* remaining susceptible [42,43,44,45]. It is important to note that the methicillin resistance and linezolid resistance of *S. aureus* occur by different mechanisms, with methicillin resistance occurring as a result of the mutations to the *mecA* gene and linezolid resistance most commonly reported to be mediated by the enzyme Cfr methyltransferase [46,47,48]. In addition to the clinical relevance of MRSA, investigating the efficacy of MRSA ATCC 33591 may provide insights into other mechanisms that might affect the efficacy of the oxazolidinones against methicillin-resistant strains or potential cross-resistance mechanisms that may be induced upon oxazolidinone treatment.

**Figure 1:**
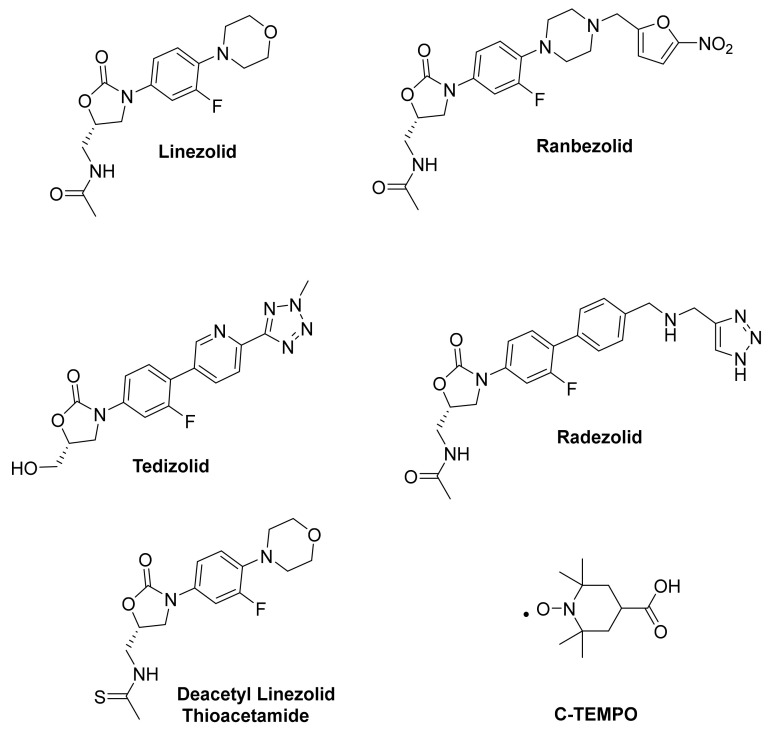
Compounds evaluated in this study. The oxazolidinone antibiotics linezolid, ranbezolid, tedizolid, radezolid, and deacetyl linezolid thioacetamide and the nitroxide C-TEMPO, which is a dispersal agent.

Previous studies assessing the anti-biofilm activity of oxazolidinones have mainly focused on biofilm inhibition, with only a few studies investigating the activity of oxazolidinones for eradicating established biofilms [49,50,51]. These inhibition and eradication studies have focused on Gram-positive species such as *Enterococcus faecalis* and methicillin-sensitive *S. aureus* and very little is known about their ability to eradicate MRSA biofilms [52]. A study by Becerra and colleagues found that linezolid reduced 98.6% and 99.8% of biofilms from MRSA ATCC 4330 and a clinical MRSA isolate at 20 µg/mL, respectively [53]. Another study by Wong and co-workers evaluated the antibiofilm activity of linezolid against MRSA USA300-0114 [54]. It was found that linezolid killed 80% of the biofilm at 256 µg/mL. There are limited studies that have investigated the impact of combination therapies with oxazolidinones on MRSA biofilms. A study by Coenye and co-workers investigated the treatment of MRSA biofilms using the quorum-sensing inhibitor Hamamelitannin in combination with antibiotics. Hamamelitannin was found to increase the susceptibility of the Mu50 biofilms to linezolid and the combination killed approximately 50% more of the biofilm in comparison to linezolid alone [55]. This highlights the potential of combination therapy as a strategy to enhance anti-biofilm activity. In this work, we examine the extent to which some of the oxazolidinone-based antibiotics (Figure 1) can eradicate MRSA biofilms and determine if their anti-biofilm activity could be further enhanced in combination with the biofilm dispersal agent C-TEMPO.

## 2. Results

### 2.1. The Oxazolidinones Have Inherent Biofilm Activity against S. aureus Biofilms

The susceptibility to the five oxazolidinone antibiotics and C-TEMPO was determined against MRSA ATCC 33591 grown in vitro in planktonic and biofilm cultures (Table 1). All other oxazolidinones displayed better anti-staphylococcal activity than linezolid (1–2-fold higher) against planktonic MRSA cells. Overall, tedizolid showed the best activity against planktonic MRSA, with an MIC of 0.5 µg/mL, while ranbezolid, radezolid, deacetyl linezolid thioacetamide performed similarly, with an MIC of 1 µg/mL. These results were expected for MRSA ATCC 33591 [50,56,57,58]. While all oxazolidinone antibiotics tested were potent against planktonic MRSA, the opposite effect was seen against MRSA biofilms.

Traditionally, within the literature, the minimum biofilm eradication concentration (MBEC) is defined as the concentration required to kill 99.9% (3-log reduction) of the biofilm in comparison to untreated controls (termed MBEC_99.9_ in Table 1). Radezolid and deacetyl linezolid thioacetamide had MBEC_99.9_ values that matched their MIC suggesting potent anti-biofilm activity. Linezolid and ranbezolid killed 99.9% of the biofilm at a 4-fold higher concentration than their MIC, while tedizolid eradicated MRSA biofilms at 2 × MIC. These results indicate that the oxazolidinone class have some inherent antibiofilm activity. However, when considering complete (100%) biofilm eradication (MBEC_C_, Table 1) only ranbezolid afforded the eradication of established MRSA ATCC 33591 biofilms at an 8-fold higher concentration in comparison to its MBEC_99.9_. All other oxazolidinones tested had MBECc values over 128–1024 times their respective MBEC_99.9_ values. These results indicate that MRSA biofilms were highly tolerant to these drugs, and the differences observed between the MBECc and the MBEC_99.9_ may indicate the presence of persister cells within the biofilm or some other type of biofilm tolerance. This also further highlights the challenge of only evaluating planktonic susceptibility in antibiotic development.

### 2.2. C-TEMPO Coadministration Improves the Susceptibility of S. aureus Biofilms to Ranbezolid

**Figure 2:**
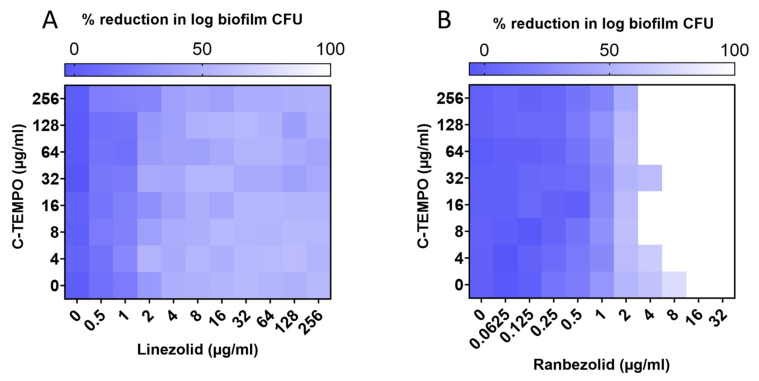
Heat plot of MRSA ATCC 33591 biofilm eradication checkerboard assay with (**A**) linezolid in combination with C-TEMPO and (**B**) ranbezolid in combination with C-TEMPO.

Previous studies have established that nitroxides can induce the dispersal of biofilms [59,60] We sought to investigate whether the nitroxide C-TEMPO could synergistically increase the susceptibility of MRSA biofilms to oxazolidinone antibiotics. As ranbezolid had the lowest MBEC value, and linezolid, tedizolid, deacetyl linezolid thioacetamide and radezolid performed similarly against MRSA biofilms, linezolid and ranbezolid were chosen as the representative antibiotics in this experiment. For linezolid, we wanted to examine whether we could achieve 100% eradication of MRSA biofilms with the coadministration of C-TEMPO. For ranbezolid, we wanted to investigate whether we could increase its susceptibility with C-TEMPO to decrease the concentration needed to achieve 100% eradication. As expected, C-TEMPO alone did not possess any antibacterial or antibiofilm activity against MRSA (MIC = 4096 µg/mL and MBEC = >2048 µg/mL) (Table 1). The coadministration of C-TEMPO at 4–256 µg/mL with linezolid at 0.5–256 µg/mL did not change the susceptibility of MRSA biofilms to linezolid (Figure 2A). The coadministration of C-TEMPO however with ranbezolid resulted in improved MBEC values (Figure 2B). In particular, C-TEMPO concentrations between 4 and 256 µg/mL reduced the MBEC_C_ value for ranbezolid by 2-fold (to 8 µg/mL). The greatest activity enhancement was achieved with 8 µg/mL of C-TEMPO, which reduced ranbezolid’s MBEC_C_ to 4 µg/mL (4-fold improvement). At this concentration, ranbezolid alone eradicates 99.9% of biofilm MRSA cells (Table 1); with C-TEMPO, it can eradicate the whole biofilm.

The dose reduction index (DRI) was then calculated to determine the extent to which the concentration of ranbezolid and C-TEMPO in the combination could be reduced to achieve 100% eradication in comparison to the MBEC achieved with the single agents (Table 2). To assess the synergistic effects between ranbezolid and C-TEMPO the fractional eradication concentration index (ΣFBEC) was calculated. At an ΣFBEC of 0.25 C-TEMPO and ranbezolid were determined to interact synergistically.

**Figure 3:**
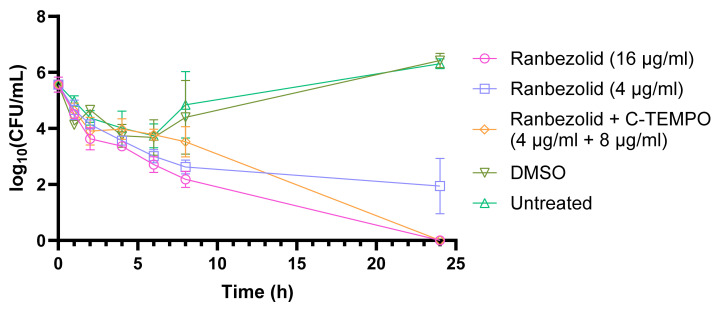
Time-kill curves of ranbezolid at its Minimum Biofilm Eradication Concentration (MBEC_C_), the synergistic combination of ranbezolid with C-TEMPO and the concentration of ranbezolid when used alone at the same concentration as the synergy experiment. Dimethyl sulfoxide (DMSO) concentration used in the experiment was 2.7% *v*/*v*. The results are shown as means ± standard error of at least two independent experiments.

Time-kill assays were performed to evaluate the activity of ranbezolid alone and in combination with C-TEMPO against established MRSA biofilms over a 24 h treatment period (Figure 3). Ranbezolid administered at its MBEC_C_ value (16 µg/mL) and the synergistic combination of ranbezolid with C-TEMPO (4 µg/mL + 8 µg/mL) performed similarly over the course of the experiment and after 24 h resulted in 100% reduction in log biofilm CFU. In contrast, when ranbezolid was used alone at the same concentration as in the synergy experiment (4 µg/mL), only a ~3-log reduction was observed after 8 h which did not improve after 24 h of treatment. Interestingly, ranbezolid administered at its MBECc value (16 µg/mL) acted much faster than the combination treatment. After 8 h of treatment, ranbezolid at 16 µg/mL achieved a 3-log reduction, but the combination treatment only achieved a 2-log reduction at the same time point. The untreated biofilms or biofilms treated with DMSO (vehicle control) maintained high numbers of viable bacteria at all time points tested. These results support the findings from the MBEC and checkerboard assays above, that ranbezolid and C-TEMPO act synergistically on biofilms.

## 3. Discussion

The mode of action of oxazolidinones against planktonic cells is well understood, while the mechanism of oxazolidinones against biofilms is currently unknown. Our study has shown that while linezolid, tedizolid, radezolid and deacetyl linezolid thioacetamide could not completely eradicate established MRSA biofilms in vitro, all oxazolidinones tested displayed some inherent antibiofilm activity (as MBEC_99.9%_ values were similar to respective MICs, Table 1). This class of oxazolidinone antibiotics inhibit protein synthesis by binding to the 50S ribosome. Our working model is that they also inhibit protein synthesis within the biofilm. It has been reported that *S. aureus* contains the intercellular adhesion (*ica*) operon (containing five genes: *icaA*, *icaB*, *icaC*, *icaD*, and *icaR*), which is responsible for slime production, and encodes the production of polysaccharide intercellular adhesion [61] It has been demonstrated that, in *S. aureus* biofilms, linezolid interferes with the transmembrane protein IcaA and the surface protein IcaB, which results in the invagination of the cell membrane and inhibits biofilm production [62]. However, the mechanism by which this interference occurs has not yet been reported. The *icaA* gene encodes the enzyme *N*-acetylglucosaminyltransferase, which mediates cell-to-cell adhesion and polysaccharide synthesis, thus enabling the formation of a 3D-structured biofilm [63]. The *icaB* gene encodes an enzyme which is responsible for the deacetylation of the polysaccharide before it binds to the cell surface [64]. The *icaD* gene is involved with the events leading to the expression of a capsular antigen called capsular polysaccharide/adhesin, which is involved in the first stage of biofilm formation where bacteria initially adhere to a surface [65,66]. While biofilms have been shown to be more resistant to antibiotics than their planktonic counterparts, antibiotics such as ciprofloxacin have been shown to diffuse through the extracellular polymeric substance (EPS) [67,68]. While it is unclear as to whether the oxazolidinones can diffuse through the EPS, it is possible that like ciprofloxacin the oxazolidinones may also diffuse through the EPS. Upon diffusing they may also interact with one of the components in the EPS. The most common components in the EPS for *S. aureus* biofilms are proteins, poly-*N*-acetyl-β-(1–6)-glucosamine and extracellular DNA and the reduction of one or all of these components may result in reduced biofilm adhesion and destruction of the biofilm [69]. Extracellular RNA was found to be a key component of *Pseudomonas Aeruginosa* biofilms and its reduction was associated with increased cell death and cell lysis [70]. Extracellular RNA has also been shown to be a crucial component of the EPS for MRSA biofilms [71]. Given the mechanism of action of the oxazolidinones, they may be able to indirectly effect matrix degradation by interacting with the extracellular RNA leading to increased cell death and cell lysis. The diffused oxazolidinones may also kill biofilm cells via their known planktonic mechanism of action. Due to the complex nature of the biofilm microenvironment, the persister cells present in the population are not killed following antibiotic treatment, leading to incomplete eradication [72,73,74]. These findings may explain why oxazolidinones have inherent anti-biofilm activity. It is suspected that linezolid and oxazolidinones interact with a protein that prevents cell-to-cell adhesion, which leads to reduced biofilm production. However, the cells that are attached to the surface are left behind as a different protein is involved in their attachment to the surface.

**Figure 4:**
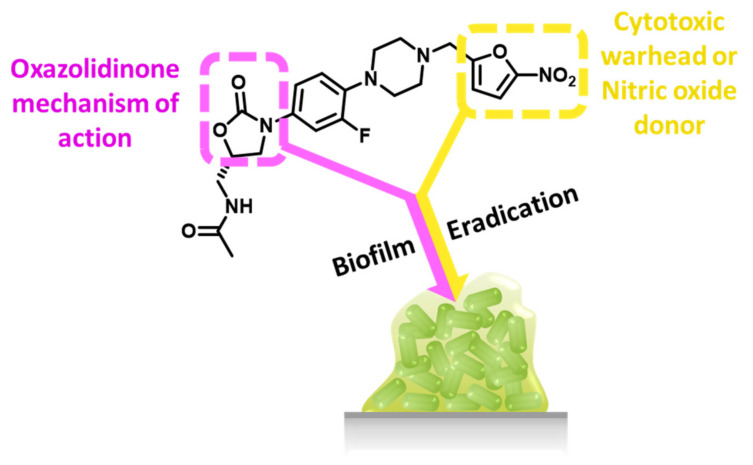
A suggested rationale for ranbezolid’s biofilm eradication properties.

Our findings show that ranbezolid was the only oxazolidinone to eradicate *S. aureus* biofilms completely (100%). The physiochemical properties of the oxazolidinones were calculated using SwissADME’s online server (Table 3) [75] to determine if the results obtained were a result of physiochemical differences. The ability of ranbezolid to completely eradicate *S. aureus* biofilms does not seem to be explained by its physiochemical properties, as all the oxazolidinones were similar. It is suspected that the difference in activity may lie in its structure. In comparison to linezolid, the structure of ranbezolid replaces the morpholine moiety with a piperazine ring attached to a nitrofuran via a methylene linker. Our previous work showed the piperazinyl oxazolidinone derivative is not able to eradicate *S. aureus* biofilms completely [59]. Therefore, it is suspected that the key to ranbezolid’s potent antibiofilm activity lies in its nitrofuran ring. Nitrofuran-based antibiotics such as nitrofurantoin and nitrofurazone have been reported to have activity against *S. aureus* [76,77]. The mechanism of action of these compounds involves nitroreductases. Type II nitroreductases catalyse the reduction of the nitro group into reactive nitroso and hydroxylamine derivatives, and from there, the nitroso can be converted into nitric oxide which has been shown to disperse MRSA biofilms [78,79,80,81,82]. Type I nitroreductases catalyse the nitro moiety to nitroanion free radical, which is toxic to cells [78,82,83,84,85,86]. We hypothesize that ranbezolid functions as a ‘dual-warhead’ drug to eradicate biofilms and acts via the oxazolidinone mode of action and as either a nitric oxide donor or cytotoxic drug because of its nitrofuran ring (Figure 4). While the exact mechanism of action of nitroxide hybrid drugs is currently unknown [59,60,87], nitroxides are considered to be nitric oxide mimics. Combination treatment was then used to explore whether the susceptibility of ranbezolid and linezolid could be enhanced with the nitroxide C-TEMPO.

It was hypothesised that when the nitroxide C-TEMPO was used in combination with ranbezolid and linezolid, it would lead to increased effectiveness in the eradication of MRSA biofilms, as the biofilm would be dispersed into planktonic cells which are susceptible to the antibiotic treatment. For ranbezolid, our findings (Figure 2 and Figure 3) show a modest improvement (4-fold reduction) in biofilm eradication, whereas linezolid did not eradicate the biofilm in co-administration with C-TEMPO. We have previously found that a hybridisation strategy where linezolid was covalently linked to C-TEMPO could completely eradicate (100%) in vitro MRSA biofilms at 160 µg/mL. In comparison to the MBEC reported in this work, this represents a 6.4-fold decrease. This suggests that strategies need to be tailored for each drug to increase the activity of oxazolidinone-based antibiotics against MRSA biofilms.

## 4. Materials and Methods

### 4.1. Bacterial Strains and Culture Conditions

Methicillin-resistant *Staphylococcus aureus* (MRSA) strain ATCC 33591 was grown routinely in lysogeny broth (LB) with shaking (200 rpm) at 37 °C. MIC assays were conducted in Mueller-Hinton (MH) medium (OXOID, Thermo Fisher, Scoresby, Australia). Biofilms were grown in Tryptic Soy Broth (TSB) (BD Micro, Bacto Laboratories, Mt Pritchard, Australia).

### 4.2. Nitroxide and Antibiotics

Linezolid and tedizolid were obtained from Merck, radezolid, deacetyl linezolid thioacetamide and C-TEMPO were obtained from Novachem. Ranbezolid was synthesized in house following a literature procedure which has been detailed in the supplementary information [59,88]. Stock solutions of all compounds were prepared in DMSO and stored at −20 °C. Working stocks were prepared on the day of experimentation in either MH or TSB.

### 4.3. Nitroxide and Antibiotic MIC Assay

The MICs for linezolid, tedizolid, radezolid, deacetyl linezolid thioacetamide, ranbezolid and C-TEMPO were determined using the broth microdilution method as outlined by the 2015 (M07-A10) Clinical and Laboratory Standards Institute (CLSI) [89]. Briefly, using a 96-well plate, each compound was serially diluted in two-fold dilutions to give a final volume of 100 µL. Then, 100 µL of a 5 × 10^6^ bacterial colony forming units (CFU)/mL solution that was prepared from fresh cultures in MH was used to inoculate each well. Untreated growth control, media only and DMSO (2.6–3.4% *v*/*v*) controls were also included. The MIC for each compound was determined as the lowest concentration that prevented visible growth after 18 h of incubation at 37 °C in accordance with the EUCAST guidelines.

### 4.4. Biofilm Culture Using the Minimum Biofilm Eradication Concentration Device

An unmodified minimum biofilm eradication concentration (MBEC) device (formerly known as the Calgary Biofilm device) purchased from Innovotech Inc. (Edmonton, AB, Canada, cat no. 19111) was used to grow MRSA biofilms. The device consists of a standard flat-bottom 96-well plate and a lid attached to 96 pegs. Biofilm cultures were prepared as previously described in the literature [90]. Briefly, a starting inoculum of 1 × 10^7^ in TSB was prepared from overnight cultures, and 130 µL was used to inoculate each well in the flat-bottom 96-well plate. The peg lid was inserted into inocula-containing wells, and the MBEC device was incubated for 24 h with shaking at 150 rpm at 37 °C in relative humidity.

### 4.5. Minimum Biofilm Eradication Concentration (MBEC) Assays

Biofilms were established as detailed above. To determine the MBECs for all compounds, peg lids containing established biofilms were removed and rinsed for 2 s in phosphate-buffered saline (PBS) (in a 96-well plate, 130 µL per well) to remove any loosely adhered bacteria. The rinsed peg lid was then transferred to a new flat-bottom 96-well plate for treatment (challenge plate). The challenge plate contained 2-fold serial dilutions of linezolid (with a concentration range of 1024 to 0.5 µg/mL), tedizolid (with a concentration range of 1024 to 0.125 µg/mL), radezolid (with a concentration range of 1024 to 0.03125 µg/mL), deacetyl linezolid thioacetamide (with a concentration range of 1024 to 0.03125 µg/mL), ranbezolid, (with a concentration range of 1024 to 0.0625 µg/mL) and C-TEMPO (with a concentration range of 2048 to 1 µg/mL). An untreated growth control containing TSB and DSMO-treated (vehicle control) matching the highest compound concentration (2.6–3.4% *v*/*v*) in TSB medium was also included. The MBEC device was then incubated for 24 h by shaking at 150 rpm at 37 °C in relative humidity. The lid was removed from the challenge plate and rinsed in PBS (in a 96-well plate, 200 µL per well) for 2 s. The now rinsed lid was transferred to a new flat-bottom 96-well plate containing PBS (200 µL per well) for CFU enumeration. The MBEC device was then sonicated for 30 min (<20 °C) according to the Innovotech MBEC Assay^®^ procedural manual [91] to aid the transfer of any remaining viable biofilm cells to the recovery medium. The peg lid was discarded, and the biofilm-recovered bacteria were serially diluted 10-fold and spotted onto LB agar plates for CFU enumeration. The MBEC for each compound was determined as the lowest concentration that resulted in no recoverable CFU after 18 h growth and (complete eradication/MBEC_C_) and as a 3-log reduction (99.9% eradication) compared to untreated (growth) controls (MBEC_99.9_).

### 4.6. Checkerboard MBEC Assay

The checkerboard MBEC assays were performed as described above for MBEC assays, with the following modifications. The challenge plate consisted of 2-fold serial dilutions of linezolid (with a concentration range of 256 to 0.5 µg/mL) with C-TEMPO (with a concentration range of 256 to 4 µg/mL), and ranbezolid, (with a concentration range of 1024 to 0.0625 µg/mL) with C-TEMPO (with a concentration range of 256 to 4 µg/mL).

After CFU enumeration, the fractional biofilm eradication concentration index (FBEC) was determined as the eradication concentration of the combination divided by the concentration of the compound alone (Table 2). The combination index was determined from the lowest concentrations that resulted in no recoverable CFU after 18 h growth. With this method, synergy was defined as an FBEC index (ΣFBEC) of ≤0.5, additivity was defined as between 0.5 and 1, indifference was defined as between 1 and 2, and antagonism was defined as >2. The dose reduction index (DRI) was determined from the lowest concentrations that resulted in no recoverable CFU after 18 h growth (Table 2).

(1) $$\Sigma \mathrm{FBEC}={\mathrm{FBEC}}_{A}+{\mathrm{FBEC}}_{B}=\frac{C_{A}}{{\mathrm{MBEC}}_{A}}+\frac{C_{B}}{{\mathrm{MBEC}}_{B}}$$

Equation (1) is FBEC index calculation using the eradication concentration of antibiotic (C_A_) and C-TEMPO (C_B_) in combination and the concentrations of the antibiotic (MBEC_A_) and C-TEMPO (MBEC_B_).

(2) $$\mathrm{DRI}=\frac{\mathrm{MBEC}\;\mathrm{of}\;\mathrm{compound}\;\mathrm{alone}}{\mathrm{MBEC}\;\mathrm{of}\;\mathrm{compound}\;\mathrm{in}\;\mathrm{combination}}$$

Equation (2) is dose reduction index calculation using the MBEC of each compound alone and in combination.

### 4.7. Biofilm Time Kill Assay

The biofilm time kill assays were performed as described above for MBEC assays, with the following modifications. The challenge plate contained either ranbezolid alone or in combination with C-TEMPO. For ranbezolid alone, the concentrations used were 16 µg/mL and 4 µg/mL. For cotreatment, ranbezolid was used at 4 µg/mL with 8 µg/mL of C-TEMPO. At the specified time points of 0 (before peg lid is placed into the wells of the challenge plate), 1, 2, 4, 6, 8 and 24 h of incubation in the challenge plate, pegs were removed from the wells and snapped off the peg lid. The peg lid was placed back into the challenge plate and incubated until the next time point. The snapped pegs were rinsed in PBS (in a 96-well plate, 200 µL per well) for 2 s and transferred to a new flat-bottom 96-well plate containing PBS (200 µL per well) for CFU enumeration. The microtiter plate was then sonicated for 30 min (<20 °C) to aid the transfer of any remaining viable cells to the recovery medium. The pegs were discarded, and the remaining biofilm-recovered bacteria were serially diluted 10-fold and spotted onto LB agar plates for CFU enumeration.

## 5. Conclusions

In conclusion, we have shown that the oxazolidinones used in this study have inherent anti-biofilm activity, but with the exception of ranbezolid, they cannot completely eradicate biofilms in vitro. Ranbezolid was shown to be a promising biofilm eradication agent and warrants further investigation into its mechanism of action and use as an anti-biofilm agent. Further preclinical and clinical studies are required for the ranbezolid and C-TEMPO combination, particularly with chronic patient cohorts, which has been identified to be lacking in available clinical trial data. The findings of this study also merit further investigation of other ranbezolid combination therapies to eradicate MRSA biofilms, as well as the development of new oxazolidinone biofilm eradication agents that are based on ranbezolid. This work also serves as the first time (to the best of our knowledge) that the oxazolidinones in this study, with the exception of linezolid, have been tested for their ability to eradicate MRSA biofilms. It was also the first time that ranbezolid had been tested for its ability to eradicate any biofilm and the first time deacetyl linezolid thioacetamide had been tested against biofilms.

## Figures and Tables

**Table 1:** Minimum inhibitory concentration (MIC) and minimum biofilm eradication concentrations (99.9% and complete) for oxazolidinone drugs and the nitroxide biofilm dispersal agent C-TEMPO against MRSA ATCC 33591.

Treatment	MIC (µg/mL)	MBEC_99.9_ ^a^ (µg/mL)	MBEC_C_^b^ (µg/mL)
Linezolid	2	8	>1024
Tedizolid	0.5	4	>512
Ranbezolid	1	2	16
Radezolid	1	1	>1024
Deacetyl Linezolid Thioacetamide	1	1	>1024
C-TEMPO	4096	>2048	>2048

^a^ MBEC-99.9, the concentration for 99.9% biofilm eradication in comparison to untreated (growth) controls. ^b^ MBEC-complete, the concentration for 100% biofilm eradication in comparison to untreated (growth) controls.

**Table 2:** Minimum biofilm eradication concentration (MBEC-complete), fractional biofilm eradication concentration (FBEC), dose reduction index (DRI) for ranbezolid and C-TEMPO against MRSA ATCC 33591.

Compound	MBEC_C_ (µg/mL)	FBEC	DRI
Ranbezolid	16	0.25	4
C-TEMPO	>2048	0.0039	256
Ranbezolid + C-TEMPO	-	0.2539 ^a^	-

^a^ Fractional biofilm eradication concentration index (ΣFBEC) derived by the sum of both FBECs.

**Table 3:** In silico molecular properties prediction for the five oxazolidinones using SwissADME’s online server [75].

Compound	MW ^a^ (g/mol)	logP ^b^	H Bond Donors ^c^	H Bond Acceptors ^d^	Rotatable Bonds ^e^	TPSA ^f^ (Å^2^)
Linezolid	337.35	1.26	5	1	5	71.11
Tedizolid	370.34	1.47	8	1	4	106.26
Ranbezolid	461.44	1.24	8	1	8	124.08
Radezolid	438.45	2.07	7	3	9	112.24
Deacetyl Linezolid Thioacetamide	353.41	1.95	4	1	5	86.13

^a^ Molecular weight (<500). ^b^ Logarithm of partition coefficient between n-octanol and water (<5). ^c^ Number of hydrogen bond donors (<5). ^d^ Number of hydrogen bond acceptors (<10). ^e^ Rotatable bonds (<10). ^f^ Topological polar surface area (<140).

## Data Availability

Data are contained within the article and Supplementary Materials.

## Supplementary Materials

The following supporting information can be downloaded at: https://www.mdpi.com/article/10.3390/antibiotics12121706/s1, Scheme S1: Synthesis of ranbezolid, Figure S1: HPLC of (S)-N-((3-(3-fluoro-4-(4-((5-nitrofuran-2-yl)methyl)piperazin-1-yl)phenyl)-2-oxooxazolidin-5-yl)methyl)acetamide (6, ranbezolid), Table S1: % Reduction in biofilm CFU (compared to untreated controls) for the oxazolidinones.
