# Supplementary material for: In Vitro Activities of Oxazolidinone Antibiotics Alone and in Combination with C-TEMPO against Methicillin-Resistant Staphylococcus aureus Biofilms
Source: Antibiotics (Basel). 2023 Dec 7;12(12):1706. doi: 10.3390/antibiotics12121706 (PMC10741017; doi:10.3390/antibiotics12121706)
Supplement: Supplementary file 1 [file antibiotics-12-01706-s001.zip › antibiotics-2737436-supplementary.pdf]

antibiotics

Supplementary file

In Vitro Activities of Oxazolidinone Antibiotics Alone and in Combination with C-TEMPO against Methicillin-Resistant *Staphylococcus aureus* Biofilms

Audrey R. N. Ndukwe [1,2,3], Jilong Qin [1], Sandra Wiedbrauk [2,3], Nathan R. B. Boase [2,3], Kathryn E. Fairfull-Smith [2,3] and Makrina Totsika [1,4,*]

[1] Centre for Immunology and Infection Control, School of Biomedical Sciences, Faculty of Health, Queensland University of Technology, Brisbane, QLD 4001, Australia; audrey.ndukwe@hdr.qut.edu.au (A.R.N.N.); jilong.qin@qut.edu.au (J.Q.)
[2] School of Chemistry and Physics, Queensland University of Technology, Brisbane, QLD 4001, Australia; sandra.wiedbrauk@qut.edu.au (S.W.); nathan.boase@qut.edu.au (N.R.B.B.); k.fairfull-smith@qut.edu.au (K.E.F.-S.)
[3] Centre for Materials Science, Queensland University of Technology, Brisbane, QLD 4001, Australia
[4] Max Planck Queensland Centre, Queensland University of Technology, Brisbane, QLD 4001, Australia
* Correspondence: makrina.totsika@qut.edu.au

Citation: Ndukwe, A.R.N.; Qin, J.; Wiedbrauk, S.; Boase, N.R.B.; Fairfull-Smith, K.E.; Totsika, M. In Vitro Activities of Oxazolidinone Antibiotics Alone and in Combination with C-TEMPO against Methicillin-Resistant *Staphylococcus aureus* Biofilms. *Antibiotics* **2023**, *12*, 1706. https://doi.org/10.3390/antibiotics12121706

Academic Editor: Lene Karine Vestby

Received: 9 November 2023
Revised: 4 December 2023
Accepted: 4 December 2023
Published: 7 December 2023

Copyright: © 2023 by the authors. Licensee MDPI, Basel, Switzerland. This article is an open access article distributed under the terms and conditions of the Creative Commons Attribution (CC BY) license (https://creativecommons.org/licenses/by/4.0/).

Ranbezolid synthesis

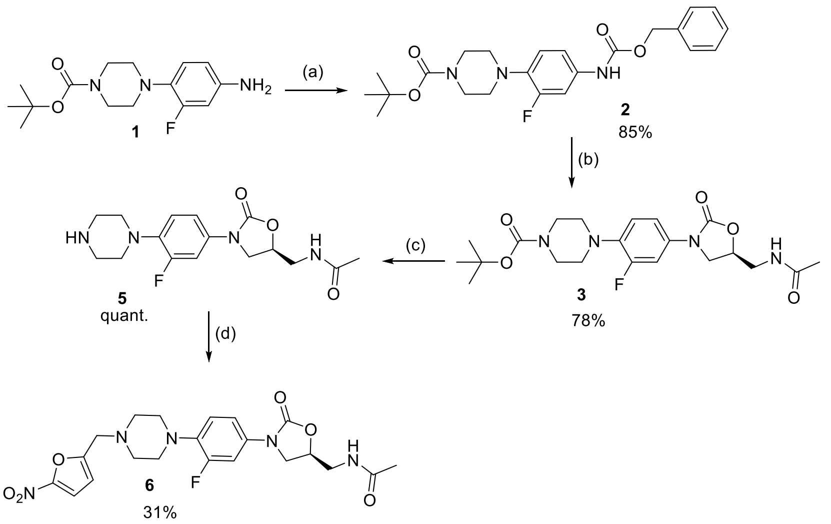

Scheme S1. Synthesis of ranbezolid. Reagents and conditions: (a) benzyl chloroformate, DIPEA, acetone, RT, 2 h; (b) (S)-1-acetamido-3-chloropropan-2-yl acetate (4), t-BuOLi, MeOH, DMF, RT, 25 h; (c) TFA, RT, 5 h; (d) 5-nitrofuran-2-carboxaldehyde, sodiumtriacetoxyborohydride, THF, molecular sieves 4 Å, RT, 26 h.

Experimental Section

Materials and instrumentation

Air-sensitive and moisture-sensitive reactions were carried under an atmosphere of argon. Anhydrous methanol and DMF were dried over molecular sieves. Anhydrous toluene and diethyl ether were dried by storage over sodium wire. Anhydrous THF was obtained from a Pure Solv solvent purification system by Innovative Technologies. Acetone was distilled on a rotary evaporator prior to use. All other reagents were used without further purification, as obtained from commercial suppliers. ^{1}H, ^{13}C, and ^{19}F NMR spectra were recorded at 600 MHz, 151 MHz, or 565 MHz respectively on a Bruker Advance 600. Samples were prepared in either $CDCl_3$, DMSO-d_6 or CD_3OD. Spectra were

referenced to residual solvent peaks: $CHCl_3$ (7.26 ppm for ^{1}H and 77.2 ppm for ^{13}C NMR spectra), DMSO (2.50 ppm for ^{1}H and 39.5 ppm for ^{13}C NMR spectra), Acetone (2.04 ppm for ^{1}H and 29.8 and 206.3 ppm for ^{13}C NMR spectra) or MeOH (3.31 ppm for ^{1}H and 49.0 ppm for ^{13}C NMR spectra). Analytical HPLC was carried out on either an Agilent Technologies HP 1100 Series HPLC system or a Dionex Ultimate 3000 system using an Interchim US5C18HQ-250/212 or an Agilent C18 column (4.6 × 150 mm, 10 µm) and with a flow rate of 1 mL/min. Column chromatography was performed using LC60A 40-63 Micron DAVISIL silica gel. Thin-layer chromatography (TLC) was performed on Merck Silica Gel 60 F254 plates. Plates were visualised under a UV lamp (254 nm) and/or developed with potassium permanganate or iodine stains. Melting points were measured on a Buchi M-565 Melting point Apparatus by the capillary method.

Synthesis of the oxazolidinone ring

Synthesis of tert-Butyl 4-(4-(((benzyloxy)carbonyl)amino)-2-fluorophenyl)piperazine-1-carboxylate (**2**)

A solution of tert-butyl 4-(4-amino-2-fluorophenyl)piperazine-1-carboxylate (**1**) (3.09 g, 10.5 mmol, 1.00 eq.), and N,N-diisopropylethylamine (DIPEA) (2.67 mL, 15.7 mmol, 1.50 eq.) in acetone (30 mL) was stirred at 0 °C. Benzyl chloroformate (8.70 mL, 30.6 mmol, 3.00 eq., 50% in toluene) was added dropwise while keeping the temperature below 0 °C. The reaction mixture was left to warm to room temperature and stirred for 2 h. Upon completion water (18 mL) and DCM (18 mL) were added, and the phases separated. The aqueous layer was extracted with DCM (3 ´ 18 mL). The combined organic layers were washed with brine and concentrated in vacuo. Silica gel chromatography (DCM/ACN, 90:10) was used to afford **2** as a light pink solid (3.82 g, 85%). m.p = 152.2 – 153.5 °C. ^{1}H NMR (600 MHz, $CDCl_3$) δ = 7.41 – 7.30 (m, 6H), 7.00 – 6.95 (m, 1H), 6.85 (t, J = 9.0 Hz, 1H), 6.77 – 6.74 (m, 1H), 5.18 (s, 2H), 3.57 (t, J = 5.1 Hz, 4H), 2.95 (t, J = 5.1 Hz, 4H), 1.48 ppm (s, 9H). HMRS (ESI): Calculated m/z for $C_{23}H_{29}FN_3O_4$ $[M+H]^+$: 430.2137, found m/z 430.2132 (Δ m/z = 1.16 ppm)

Synthesis of tert-Butyl(S)-4-[4-[5-(acetamidomethyl)-2-oxooxazolidin-3-yl]-2-fluorophenyl]piperazine-1-carboxylate (**3**)

A solution of lithium tert-butoxide (40.0 mL, 40.0 mmol, 6.00 eq., 1 M in THF) was added to a solution of benzyl carbamate **2** (2.87 g, 6.68 mmol, 1.00 eq.) in DMF (10 mL) and methanol (1.10 mL, 26.7 mmol, 4.00 eq.) under argon at 20 °C, keeping the temperature below 24 °C with an ice bath. The solution was cooled to 5 °C and a solution of (S)-1-acetamido-3-chloropropan-2-yl acetate **4** (5.18 g, 26.7 mmol, 4.00 eq.) in DMF (10 mL) was added. The resulting solution was left to stir at 24 °C for 25 h. Saturated ammonium chloride (20 mL), water (150 mL), and DCM (100 mL) was added, and the phases separated. The aqueous layer was extracted with DCM (3 ´ 300 mL). The combined organic layers were concentrated to an oil in vacuo, followed by azeotropic distillation with toluene (× 2) to remove residual DMF and afford product **3** as an off white solid (2.28 g, 78%, 96% purity). m.p = 150.8 – 153.2 °C. ^{1}H NMR (600 MHz, $CDCl_3$) δ = 7.43 (dd, J = 14.1, 2.6 Hz, 1H), 7.06 (ddd, J = 8.8, 2.6, 1.0 Hz, 1H), 6.92 (t, J = 9.1 Hz, 1H), 6.26 (t, J = 6.2 Hz, 1H), 4.76 (dddd, J = 8.8, 6.6, 5.7, 3.2 Hz, 1H), 4.01 (t, J = 9.0 Hz, 1H), 3.74 (dd, J = 9.1, 6.7 Hz, 1H), 3.68 (ddd, J = 14.7, 6.1, 3.3 Hz, 1H), 3.63 – 3.56 (m, 5H), 2.98 (t, J = 5.1 Hz, 4H), 2.01 (s, 3H), 1.47 (s, 9H), 1.24 (d, J = 8.7 Hz, 1H), 0.90 – 0.84 ppm (m, 1H). ^{13}C NMR (151 MHz, $CDCl_3$) δ = 171.1, 156.3, 154.7, 154.6, 154.3, 133.18, 133.11, 119.4, 119.3, 113.9, 113.8, 107.5, 107.4, 79.9, 71.9, 50.6, 47.6, 41.9, 29., 28.43, 23.1 ppm. HMRS (ESI): Calculated m/z for $C_{21}H_{30}FN_4O_5$ $[M+H]^+$: 437.2195, found m/z 437.2187 (Δ m/z = 1.82 ppm).

Synthesis of (S)-N-((3-(3-fluoro-4-(piperazin-1-yl)phenyl)-2-oxooxazolidin-5-yl)methyl)acetamide (**5**)

TFA (4.10 ml, 52.2 mmol, 10.0 eq.) was added to a solution of Boc-protected piperazinyl oxazolidinone **3** (2.28 g, 5.22 mmol, 1.00 eq.) in DCM (10 mL). The resulting solution was left to stir at 24 °C for 5 h 40 min. The solvent was removed in vacuo, and the resultant oil was recrystallized from methanol to give **5** as a light brown solid (quant.) m.p = 53.5 – 56.0 °C. Data obtained by ^{1}H NMR, ^{13}C NMR spectroscopy and HMRS (ESI) matched that previously reported in the literature.[29]

Synthesis of (S)-N-((3-(3-fluoro-4-(4-((5-nitrofuran-2-yl)methyl)piperazin-1-yl)phenyl)-2-oxooxazolidin-5-yl)methyl)acetamide (**6**)

Molecular sieves (4 Å) and 5-nitrofuran-2-carboxaldehyde (115 mg, 0.815 mmol, 1.30 eq.) was added to piperazinyl oxazolidinone **5** (211 mg, 0.627 mmol, 1.00 eq.) in THF (10 mL) in a round bottom flask with guard tube. The reaction mixture was left to stir at 24 °C for 1.5 h, and then sodium triacetoxy borohydride (532 mg, 2.51 mmol, 4.00 eq.) was added. The reaction mixture was left to stir at 24 °C for 26 h. DCM was added (50 mL) and the reaction mixture was filtered under vacuum. The filtrate was washed with water (100 mL x 3) and dried over sodium sulphate. The solvent was removed in vacuo. Silica gel chromatography (DCM/MeOH, 95:5) was used to afford **6** as a dark brown solid (89.8 mg, 31%). m.p = 103.7 – 105.3 °C. ^{1}H NMR (600 MHz, $CDCl_3$) δ 7.43 (dd, J = 14.2, 2.6 Hz, 1H), 7.30 (d, J = 3.6 Hz, 1H), 7.05 (ddd, J = 8.8, 2.7, 1.0 Hz, 1H), 6.91 (t, J = 9.1 Hz, 1H), 6.66 (bs, 1H), 6.25 (d, J = 6.6 Hz, 1H), 4.76 (dddd, J = 8.9, 6.7, 5.8, 3.3 Hz, 1H), 4.01 (t, J = 9.0 Hz, 1H), 3.83 (s, 2H), 3.74 (dd, J = 9.1, 6.7 Hz, 1H), 3.68 (ddd, J = 14.7, 6.1, 3.2 Hz, 1H), 3.61 (dt, J = 14.7, 6.1 Hz, 1H), 3.17 (bs, 4H), 2.84 (bs, 4H), 2.01 (s, 3H). HMRS (ESI): Calculated m/z for [M+H]+: 462.1783, found m/z 462.1785 (Δ m/z = 0.432733 ppm)

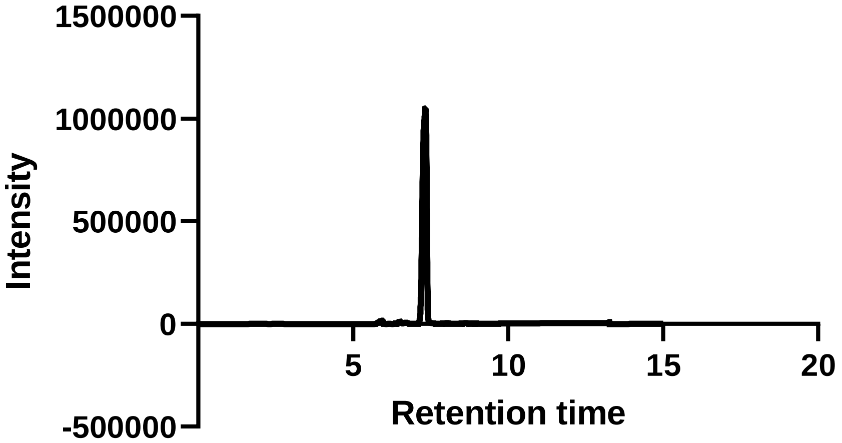

Figure S1. HPLC of (S)-N-((3-(3-fluoro-4-(4-((5-nitrofuran-2-yl)methyl)piperazin-1-yl)phenyl)-2-oxooxazolidin-5-yl)methyl)acetamide (**6, ranbezolid**)

Table S1. % Reduction in biofilm CFU (compared to untreated controls) for the oxazolidinones

Concentration (μg/mL)	% reduction in log biofilm CFU (compared to untreated controls) ± SD				
	Antibiotic				
	Linezolid	Tedizolid	Ranbezolid	Radezolid	Deacetyl linezolid thioacetamide
**0.03125**	-	-	-	-3.76 ± 11.95	-4.68 ± 3.01
**0.0625**	-	-	-5.16 ± 6.07	-4.83 ± 3.23	-3.01 ± 1.55
**0.125**	-	20.85 ± 9.67	-1.12 ± 9.05	8.10 ± 11.54	11.20 ± 6.22
**0.25**	-	48.40 ± 14.27	12.77 ± 4.87	12.32 ± 2.35	21.52 ± 11.19
**0.5**	11.45 ± 15.33	54.64 ± 7.11	15.86 ± 11.11	48.94 ± 7.07	45.73 ± 8.29
**1**	18.08 ± 8.44	48.28 ± 3.24	19.36 ± 11.59	49.97 ± 3.93	50.74 ± 1.93
**2**	37.17 ± 9.53	51.05 ± 3.05	62.52 ± 14.12	61.53 ± 5.14	49.54 ± 2.21
**4**	50.1 ± 5.57	57.12 ± 3.64	67.73 ± 14.63	57.14 ± 5.05	55.01 ± 6.39
**8**	50.96 ± 6.45	54.22 ± 1.66	81.3 ± 18.79	58.74 ± 19.18	55.84 ± 4.98
**16**	53.49 ± 3.98	53.72 ± 3.25	100 ± 0	54.36 ± 5.58	57.07 ± 4.86
**32**	55.52 ± 4.22	52.92 ± 2.61	100 ± 0	53.8 ± 5.55	54.03 ± 3.16
**64**	55.7 ± 2.41	52.88 ± 3.07	100 ± 0	52.53 ± 3.89	51.99 ± 3.66
**128**	51.11 ± 12.60	51.38 ± 2.11	100 ± 0	54.43 ± 5.73	51.19 ± 4.3
**256**	57.94 ± 4.81	51.41 ± 6.60	100 ± 0	53.82 ± 2.65	52.87 ± 4.47
**512**	46.9 ± 13.84	47.62 ± 8.64	100 ± 0	54.69 ± 4.79	50.93 ± 3.11
**1024**	53.33 ± 1.99	-	100 ± 0	50.06 ± 3.28	63.88 ± 25.72
**Average Untreated Control (log CFU/mL) ± SD**	6.32 ± 0.56	6.22 ± 0.41	6.3 ± 0.53	6.18 ± 0.32	6.18 ± 0.32
